# The Relation between Serum Phosphorus Levels and Clinical Outcomes after Acute Myocardial Infarction

**DOI:** 10.1371/journal.pone.0058348

**Published:** 2013-03-11

**Authors:** Doron Aronson, Michael Kapeliovich, Haim Hammerman, Robert Dragu

**Affiliations:** 1 Department of Cardiology, Rambam Medical Center, Haifa, Israel; 2 Rappaport Faculty of Medicine and Research Institute, Technion, Israel Institute of Technology, Haifa, Israel; University of Otago, New Zealand

## Abstract

**Background:**

Elevated serum phosphorus levels have been linked with cardiovascular disease and mortality with conflicting results, especially in the presence of normal renal function.

**Methods:**

We studied the association between serum phosphorus levels and clinical outcomes in 1663 patients with acute myocardial infarction (AMI). Patients were categorized into 4 groups based on serum phosphorus levels (<2.50, 2.51–3.5, 3.51–4.50 and >4.50 mg/dL). Cox proportional-hazards models were used to examine the association between serum phosphorus and clinical outcomes after adjustment for potential confounders.

**Results:**

The mean follow up was 45 months. The lowest mortality occurred in patients with serum phosphorus between 2.5–3.5 mg/dL, with a multivariable-adjusted hazard ratio of 1.24 (95% CI 0.85–1.80), 1.35 (95% CI 1.05–1.74), and 1.75 (95% CI 1.27–2.40) in patients with serum phosphorus of <2.50, 3.51–4.50 and >4.50 mg/dL, respectively. Higher phosphorus levels were also associated with increased risk of heart failure, but not the risk of myocardial infarction or stroke. The effect of elevated phosphorus was more pronounced in patients with chronic kidney disease (CKD). The hazard ratio for mortality in patients with serum phosphorus >4.5 mg/dL compared to patients with serum phosphorus 2.50–3.50 mg/dL was 2.34 (95% CI 1.55–3.54) with CKD and 1.53 (95% CI 0.87–2.69) without CKD.

**Conclusion:**

We found a graded, independent association between serum phosphorus and all-cause mortality and heart failure in patients after AMI. The risk for mortality appears to increase with serum phosphorus levels within the normal range and is more prominent in the presence of CKD.

## Introduction

Elevated phosphorus levels correlates with increased risk for vascular calcification in both animal models and humans with advanced chronic kidney disease (CKD). Several observational studies suggest that abnormalities of mineral metabolism contribute to cardiovascular disease (CVD) and mortality in individuals with CKD [1], [2]. In some studies the association was apparent even in healthy populations with preserved renal function [3], [4].

By contrast, several recent large studies found inconsistent associations between serum phosphorus and CVD. In the Atherosclerosis Risk in Communities Study, higher serum phosphorus was associated with increased risk for mortality but not of coronary disease [5]. An analysis of 7,259 postmenopausal women reported no associations between serum phosphorus and incident cardiovascular events during 4 years of follow-up [6].

The best known cardiovascular consequence of hyperphosphatemia is vascular calcification, which is a common complication of CKD, diabetes and aging. Vascular calcifications due to elevated phosphorus levels may potentially interact and aggravate several cardiovascular risk factors. The discordance between the results of previous studies may be explained if an elevated phosphorus level modifies the risk for specific cardiovascular events depending on individual patient susceptibility to heart failure [7], sudden arrhythmic death [8] or acute coronary events [9].

Few data are available with regard to the relationship between serum phosphorus and cardiovascular outcomes in patients with established CVD [10]. We studied the association between serum phosphorus levels and clinical outcomes in patients following acute myocardial infarction (AMI). This population represents a high risk group for mortality, heart failure and recurrent cardiovascular events.

## Methods

The study cohort consisted of patients enrolled in a prospective longitudinal observational study designed to determine predictors of postinfarction HF, with data collection starting in 2001 [11], [12]. The specific analysis of the relationship between serum phosphorus levels and clinical outcomes after AMI was not part of the initial study aims and was therefore analyzed retrospectively.

Acute myocardial infarction was diagnosed based on the Universal Definition of Myocardial Infarction [13]. Over the course of the study, the biomarkers used included creatine kinase and its myocardial fraction, MB in 2001 to 2002. From 2003 to 2009, several troponins I assays were used. For each assay, increased value for cardiac troponin was defined as a measurement exceeding the 99th percentile of a normal reference population [13].

The ethics committee of Rambam Health Care Campus reviewed and approved the study (#3176 and 0278-11-RBM). The need to obtain written informed consent was specifically waived for the following reasons: this was a retrospective study that cannot influence patient treatments or outcomes, exclusively based on data extraction from medical chart records, and it would not be feasible to get patient’s consent for access to all charts. The data were analyzed anonymously.

All patients presenting to the intensive coronary care unit with AMI were eligible for entry into the study if they had a diagnosis of AMI. Exclusion criteria were alcohol or drug dependence or abuse, active malignancy or vasculitis, active infection and rhabdomyolysis.

The primary exposure variable was the baseline serum phosphorus level, measured at the morning of the first hospital day. Baseline phosphorus levels were measured in fasting state with the posphomolybdate assay on the Siemens Dimension Clinical Chemistry System (Siemens. Newark, DE) (normal range, 2.5 to 4.5 mg/dL).

Serum calcium levels were corrected for serum albumin values using the following formula:

Corrected serum calcium (in mg/dL) = observed serum calcium+[0.8×(4 − serum albumin)], if the serum albumin level was <4 g/dL [14].

Estimated glomerular filtration rate (eGFR) was calculated based on the abbreviated MDRD (Modification of Diet in Renal Disease) study equation [15], [16]. Chronic kidney disease (CKD) was defined as a decreased eGFR <60 mL/min per 1.73 m**^2^**) [16]. High-sensitivity C-reactive protein levels were obtained on the morning of the first hospital day as previously described [17].

### Study Endpoints

The primary endpoint of the study was all-cause mortality. Secondary endpoints included rehospitalization for the development of heart failure, recurrent infarctions and ischemic stroke. Heart failure was defined as readmission to hospital for the management of heart failure (defined by the presence of new symptoms of paroxysmal nocturnal dyspnea, orthopnea or edema with one or more concurrent signs, including ventricular gallop rhythm, jugular venous distention, bilateral post-tussive rales in at least the lower third of the lung fields, elevated venous pressure, or pulmonary venous congestion on X-ray with interstitial or alveolar edema).

Ischemic stroke was defined as a neurologic deficit of sudden onset that persisted for more than 24 hours, corresponded to a vascular territory in the absence of primary hemorrhage, was not explained by other causes (e.g., trauma, infection, or vasculitis), and was corroborated by an imaging study when possible [18].

Following hospital discharge, clinical endpoint information was acquired by reviewing the national death registry and by reviewing the hospital records for major clinical events if the patient had been re-hospitalized. To confirm the diagnosis of HF, all hospital records were abstracted. Data were collected on the course and care of the patient during the hospital stay, including admission notes, consultation notes, discharge summaries, and pertinent laboratory data.

### Statistical Analysis

Continuous variables are presented as mean (SD) or medians (with interquartile ranges), and categorical variables as numbers and percentages. The baseline characteristics of the groups were compared using analysis of variance for continuous variables and by χ^2^ statistic for categorical variables.

The association between serum phosphorus level and clinical and biochemical variables was assessed by univariable linear regression for each variable separately followed by multiple linear regression with backward selection. Variables considered for inclusion in the multivariable model included: age, gender, history of hypertension, history of diabetes, smoking status estimated GFR, baseline hemoglobin, Killip class and medications.

For the association with all-cause mortality, serum phosphorus was categorized into 4 groups (<2.5, 2.5 to 3.4, 3.5 to 4.5, and >4.5 mg/dL) to avoid assuming linearity. Survival curves were constructed using the Kaplan–Meier method, and comparisons were made using the log–rank test. Stepwise Cox proportional hazards models with backward selection were used to calculate hazard ratios (HRs) and 95% confidence intervals (CI) for serum phosphorus categories. The Cox models were adjusted for age, gender, serum calcium (corrected for albumin levels), history of diabetes, hypertension, smoking status, estimated glomerular filtration rate (eGFR) [15], thrombolytic therapy coronary revascularization, anterior infarction, ST-elevation infarction, hemoglobin levels and medical therapy (beta blockers, angiotensin converting-enzyme inhibitors, loop diuretics, spironolactone and digoxin). The Cox models were also adjusted for left ventricular ejection fraction (LVEF). Similar models were used for the secondary endpoints of heart failure, recurrent infarction and stroke. The relation between the serum phosphorus as continuous variable and all-cause mortality was also assessed with the use of restricted cubic spline functions [19], which allowed us to explore nonlinear relationships between serum phosphorus and clinical outcome.

We assessed whether the effect of phosphorus levels on clinical outcome varied according to CKD status using traditional interaction testing and stratified analyses. The existence of an interaction was formally evaluated with the use of a Cox regression model incorporating terms for the main effect of serum phosphorus, the main effect of CKD (defined as eGFR <60 ml·min**^−1^**/1.73 m**^2^**), and the interaction serum phosphorus and CKD.

Serum phosphorus levels were not correlated with serum calcium levels (r = 0.02, *P* = 0.93) but were highly correlated with the calcium-phosphorus product (r = 0.95, *P*<0.0001) as previously described [3]. Therefore, only the associations of serum phosphorus and clinical outcomes were analyzed.

Differences were considered statistically significant at the 2-sided *P*<0.05 level. Statistical analyses were performed using the SPSS statistical software version 16.0 (Chicago, IL) and STATA version 11.0 (College Station, TX).

## Results

Between July 2001 and June 2009, a total of 1663 patients were recruited into the study. The majority of patients (n = 1357, 81.6%) had serum phosphorus levels within the normal range, while 167 (10.0%) patients had low serum phosphorus level and 139 (8.4%) had hyperphosphatemia.

The clinical characteristics of the patients according to baseline phosphorus levels are shown in Table 1. Patients with higher phosphorus levels were more likely to be older and females, and had higher prevalence of hypertension and diabetes, and had reduced renal function and hemoglobin and higher C-reactive protein levels. Patients with higher phosphorus levels were less likely to undergo coronary revascularization and less likely to receive thrombolytic therapy; they presented with higher Killip and had lower LVEF. Use of beta blockers, angiotensin converting-enzyme inhibitors and statins was lower among patients with elevated serum phosphorus levels.

**Table 1:** Baseline clinical characteristics.

	Serum Phosphorus (mg/dL)				
Characteristic	<2.5 (n = 167)	2.50–3.50 (n = 891)	3.51–4.50 (n = 466)	>4.5 (n = 139)	*P* Value
Age (years)	62±13	61±12	60±13	65±14	0.001
Female gender	29 (17)	152 (17)	129 (28)	53 (38)	<0.0001
Previous infarction	26 (16)	167 (19)	90 (19)	29 (21)	0.67
History of hypertension	77 (46)	430 (48)	243 (52)	89 (64)	0.003
Diabetes Mellitus	43 (26)	212 (24)	150 (32)	54 (39)	<0.0001
Current smoker	23 (14)	162 (18)	89 (19)	19 (14)	0.25
eGFR (ml·min**^−1^**/1.73 m**^2^**)	78±21	83±24	83±27	64±33	<0.0001
Hemoglobin (g/dL)	14±2	14±2	14±2	13±2	<0.0001
C-reactive protein (mg/dL)*	17.3 [4.3–48.6]	12.8 [5.0–31.9]	13.8 [6.1–41.0]	18.1 [11.1–66.7]	0.03
Serum calcium (mg/dL)	8.8±0.7	8.9±0.6	9.0±0.6	8.6±0.9	<0.0001
Calcium-phosphorus product (mg**^2^**/dL**^2^**)	18.5±3.7	27.1±3.5	35.2±3.6	46.5±9.2	<0.0001
Thrombolytic therapy	53 (32)	188 (21)	90 (19)	18 (13)	<0.0001
Coronary revascularization	94 (56)	485 (55)	233 (48)	63 (46)	0.03
Killip class >I	33 (20)	157 (18)	100 (22)	73 (53)	<0.0001
Anterior infarction	74 (43)	358 (40)	214 (46)	73 (53)	0.02
LVEF <45%	33 (20)	157 (18)	100 (22)	73 (53)	<0.0001
Left ventricular ejection fraction (%)	44±12	46±12	45±12	40±13	<0.0001
Medications					
Beta blockers	147 (89)	789 (89)	419 (90)	112 (81)	0.03
ACE inhibitors/ARBs	143 (87)	786 (88)	404 (87)	108 (79)	0.02
Anti-platelet agents	165 (99)	874 (98)	455 (98)	133 (96)	0.47
Statins	110 (66)	658 (74)	353 (76)	94 (68)	0.04

Values are expressed as number (%) of patients, mean value ± SD, or Median [Interquartile Range].

ACE = angiotensin converting enzyme; ARB = Angiotensin receptor blockers.

* Data available in 701 patients.

In a multivariable linear regression model, serum phosphorus levels were positively associated with age, female gender, history of hypertension and diabetes mellitus, eGFR, and presentation with high Killip class (Table 2).

**Table 2:** Multiple Linear Regression Analysis with Serum Phosphorus as the Dependent Variable.

Independent Variable	Regression Coefficient (SE)	95% CI	*P* value
Age (per 10 years)	−0.07 (0.02)	−0.12 to −0.03	0.001
Female gender	0.28 (0.06)	0.16 to 0.39	<0.0001
Hypertension	0.13 (0.05)	0.04 to 0.23	0.004
Diabetes	0.14 (0.05)	0.04 to 0.24	0.005
Smoker	0.16 (0.05)	0.06 to 0.25	0.001
Ln estimated GFR	−0.34 (0.06)	−0.45 to −0.23	<0.0001
Killip Class >1	0.31 (0.06)	0.21 to 0.42	<0.0001

**Figure 1:**
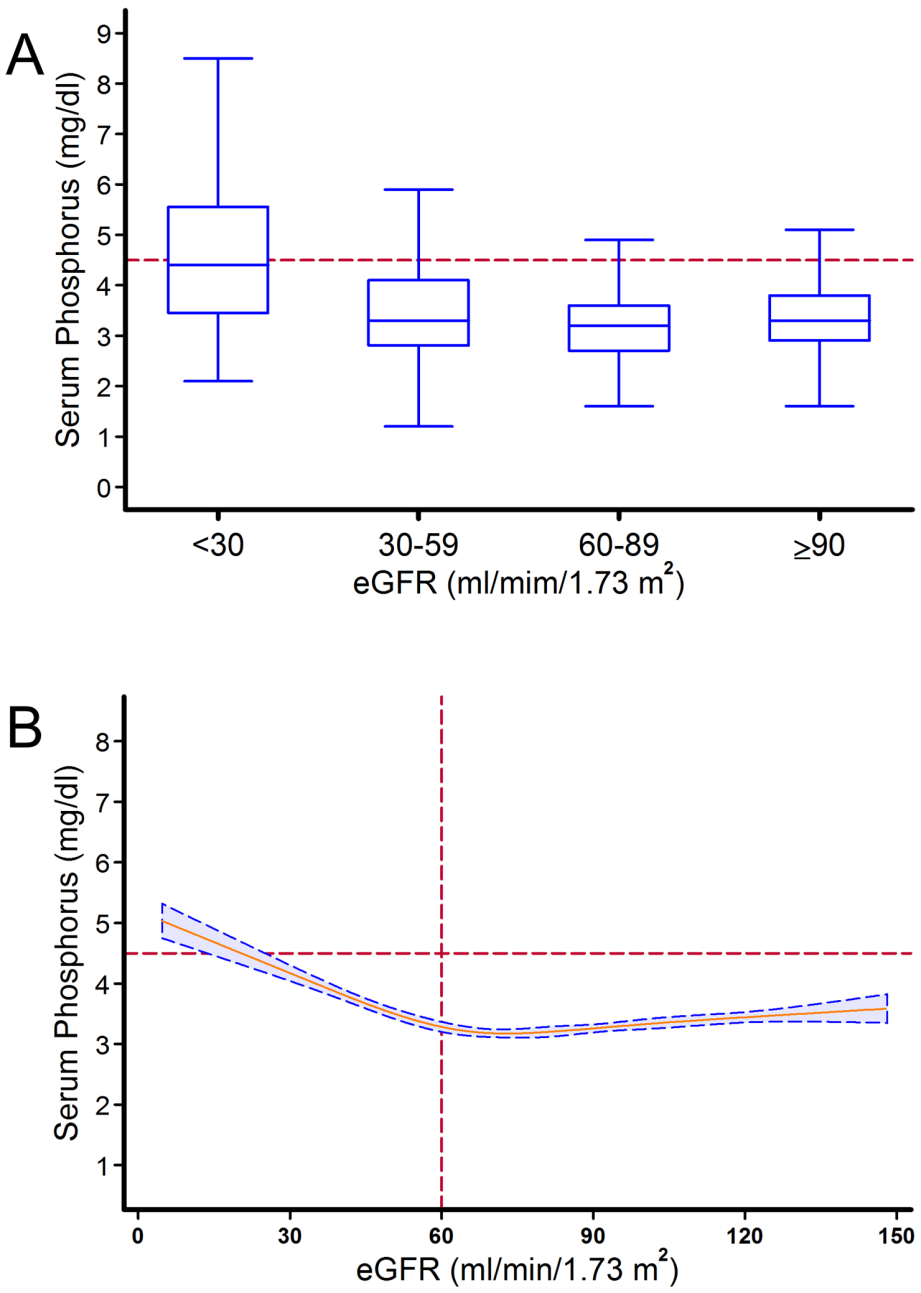
Relationship between serum phosphorus and renal function. A. Box–and–whisker plots of changes in serum phosphorus level according to the severity of renal dysfunction. The line within the box denotes the median and the box spans the interquartile range (25th to 75th percentiles). Whiskers extend from the 5th to 95th percentiles. **B.** Spline function graph of the relationship between estimated glomerular filtration rate and serum phosphorus, showing the shape of the relationship curve on a continuous basis. The blue area with dotted lines indicate the 95% confidence interval.

Figure 1A shows the relationship between serum phosphorus level and eGFR. Although serum phosphorus levels were variable within each eGFR category, there was a clear graded inverse association between the severity of renal dysfunction and serum phosphorus levels.

Hyperphosphatemia was present in 6.2%, 4.7%, 15.8% and 43.4% of patients with eGFR≥90, eGFR 60–89, eGFR 30–59 and eGFR ≤30 ml/min/1.73 m**^2^**, respectively (*P*<0.0001). However, 50.4% of patients with hyperphosphatemia had eGFR≥60. When cubic spline regression was used to explore the association between serum phosphorus and eGFR, we observed no change in serum phosphorus level at eGFR above 60 ml/min/1.73 m**^2^**, with an approximately linear increase in serum phosphorus at eGFR below 60 ml/min/1.73 m**^2^** (Figure 1B).

### Serum Phosphorus and All-cause Mortality

**Figure 2:**
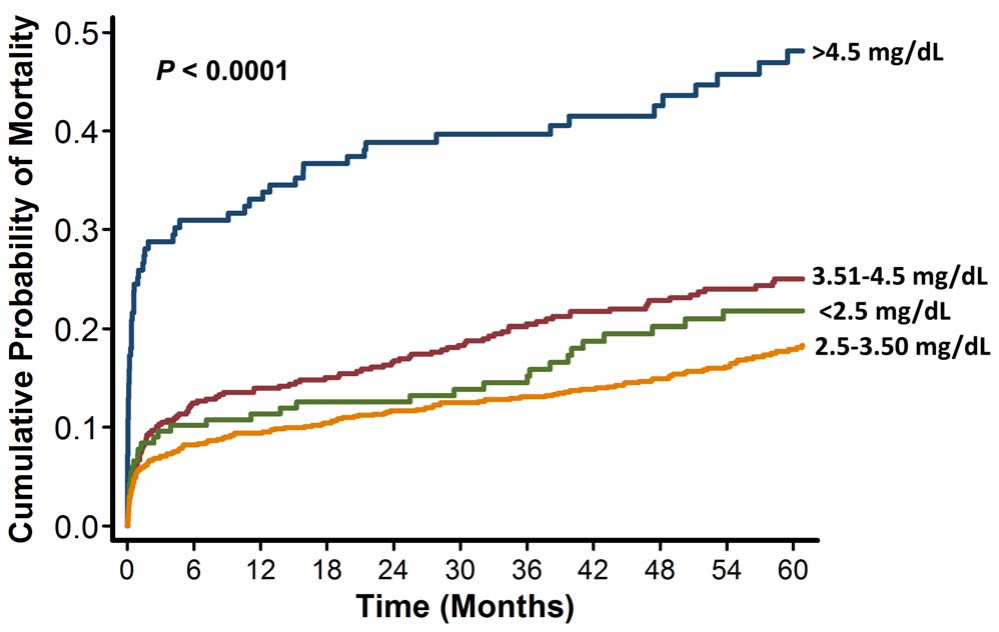
Kaplan-Meier survival plot of mortality. Patients are divided by categories of serum phosphorus. *P* values are for the overall comparison among the groups using the log rank test.

**Figure 3:**
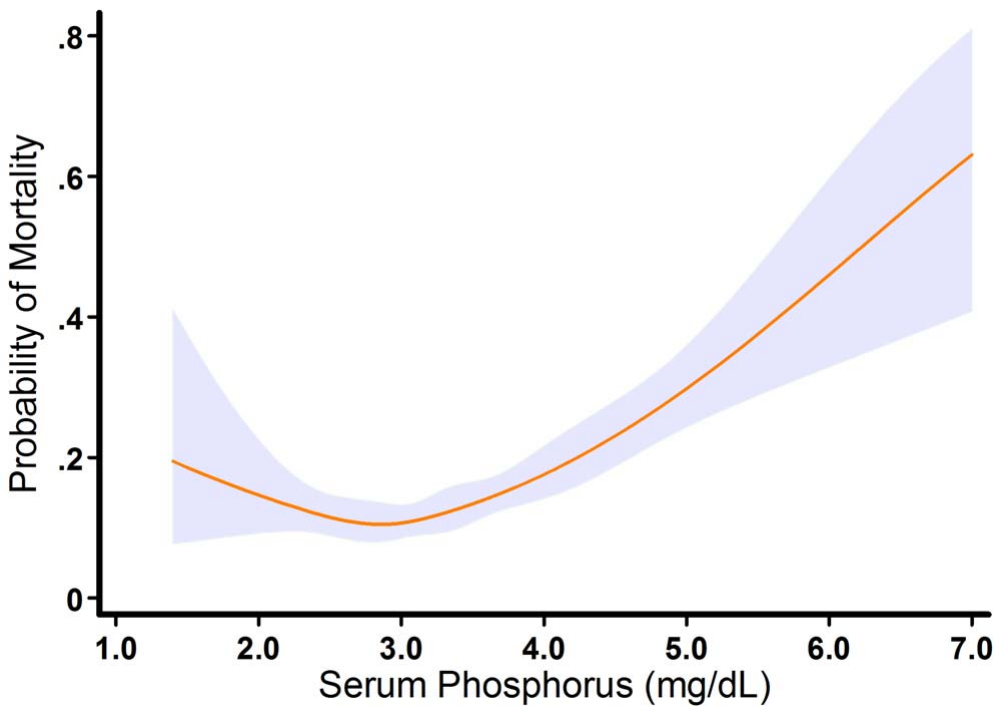
Cubic spline analysis. The plot describes the relationship between serum phosphorus as a continuous variable and the probability of mortality. The blue area indicates the 95% confidence interval.

Patients were followed between 2 and 5 years (mean 45 months). During follow up, 356 patients died (21.4%). Kaplan-Meier curves showed that the lowest mortality occurred in patients with serum phosphorus levels between 2.5–3.5 mg/dL. Mortality was higher in patients with hypophosphatemia and in patients with serum phosphorus between 3.5 and 4.0 mg/dL, with a marked increase in mortality among patients with hyperphosphatemia (Figure 2). Cubic spline analysis demonstrated that the relationship between serum phosphorus and the probability of mortality had a J-shaped relationship, increasing below 2.5 mg/dL and above 4.0 mg/dL (Figure 3).

Model 1 in Table 3 displays the results of the Cox proportional hazards model examining the relationship between serum phosphorus levels and all-cause mortality using 2.50–3.49 mg/dL as the reference category. After adjustment for other factors independently associated with mortality, the risk associated with low phosphorus level was no longer significant. The risk associated with elevated phosphorus levels was attenuated but remained statistically significant with increased risk for mortality even for patients with phosphorus levels within the normal range (Table 3).

**Table 3:** Unadjusted and Adjusted Cox's proportional Hazards Models*.

		Unadjusted			Adjusted			
Model	Events, n (%)	HR (95% CI)	*P* value	*P* trend	HR (95% CI)	*P* value	*P* trend	
All-cause mortality (Model 1)								
<2.50 mg/dL	34 (20.4)	1.28 (0.87–1.89)	0.23	<0.0001	1.24 (0.85–1.80)	0.94	0.001	
2.50–3.50 mg/dL	150 (16.8)	1.0 (Referent)	–		1.0 (Referent)	–		
3.51–4.50 mg/dL	110 (20.4)	1.49 (1.16–1.93)	0.0002		1.35 (1.05–1.74)	0.018		
>4.50 mg/dL	63 (45.3)	3.58 (2.63–4.88)	<0.0001		1.75 (1.27–2.40)	0.001		
All cardiovascular events† (Model 2)								
<2.50 mg/dL	30 (18.0)	1.06 (0.71–1.58)	0.77	<0.0001	1.04 (0.70–1.55)	0.84	0.002	
2.50–3.50 mg/dL	153 (17.2)	1.0 (Referent)	–		1.0 (Referent)	–		
3.51–4.50 mg/dL	82 (1.6)	1.06 (0.81–1.38)	0.69		1.02 (0.78–1.34)	0.80		
>4.50 mg/dL	44 (31.7)	2.64 (1.88–3.69)	<0.0001		1.73 (1.22–2.45)	0.002		
Heart failure (Model 3)								
<2.50 mg/dL	18 (10.8)	1.11 (0.66–1.87)	0.70	<0.0001	0.99 (0.59–1.66)	0.96	0.03	
2.50–3.50 mg/dL	90 (10.1)	1.0 (Referent)	–		1.0 (Referent)	–		
3.51–4.50 mg/dL	52 (11.2)	1.16 (0.82–1.65)	0.36		1.05 (0.74–1.49)	0.45		
>4.50 mg/dL	33 (23.7)	3.21 (2.13–4.85)	<0.0001		1.79 (1.17–2.73)	0.007		
Myocardial infarction (Model 4)								
<2.50 mg/dL	12 (7.2)	1.01 (0.55–1.89)	0.96	0.22	1.01 (0.55–1.88)	0.97	0.12	
g/dL	62 (7.0)	1.0 (Referent)	–		1.0 (Referent)	–		
3.51–4.50 mg/dL	37 (8.0)	1.12 (0.74–1.70)	0.58		1.19 (0.79–1.80)	0.41		
>4.50 mg/dL	14 (10.0)	1.61 (0.87–2.99)	0.30		1.57 (0.87–2.82)	0.14		
Stroke (Model 5)								
<2.50 mg/dL	5 (3.0)	0.92 (0.32–2.66)	0.88	0.42	0.76 (0.44–3.05)	0.76	0.44	
g/dL	23 (2.5)	(Referent)	–		(Referent)	–		
3.51–4.50 mg/dL	9 (1.9)	0.73 (0.34–1.58)	0.42		0.65 (0.61–3.46)	0.29		
>4.50 mg/dL	7 (5.0%)	2.02 (0.87–4.70)	0.10		1.46 (0.61–3.76)	0.40		

* All models were adjusted for age, gender, eGFR, hemoglobin, previous infarction, hypertension, diabetes mellitus, smoking, baseline hemoglobin, serum calcium, ST-elevation infarction, Killip class, coronary revascularization, LVEF.

† Heart failure, myocardial infarction or stroke.

### Secondary Endpoint

The relationship between serum phosphorus and cardiovascular events had a similar pattern to that of mortality, with increased risk for events in patients with hyperphosphatemia (Table 3, Model 2). Additional analyses for specific secondary endpoints suggested that the increased risk for cardiovascular events was mainly driven by the heart failure endpoint (Table 3, Model 3), while the relationship between elevated phosphorus level and reinfarction (Table 3, Model 4) and stroke (Table 3, Model 5) were not statistically significant.

### Impact of Serum Phosphorus and Renal Function on Mortality

**Figure 4:**
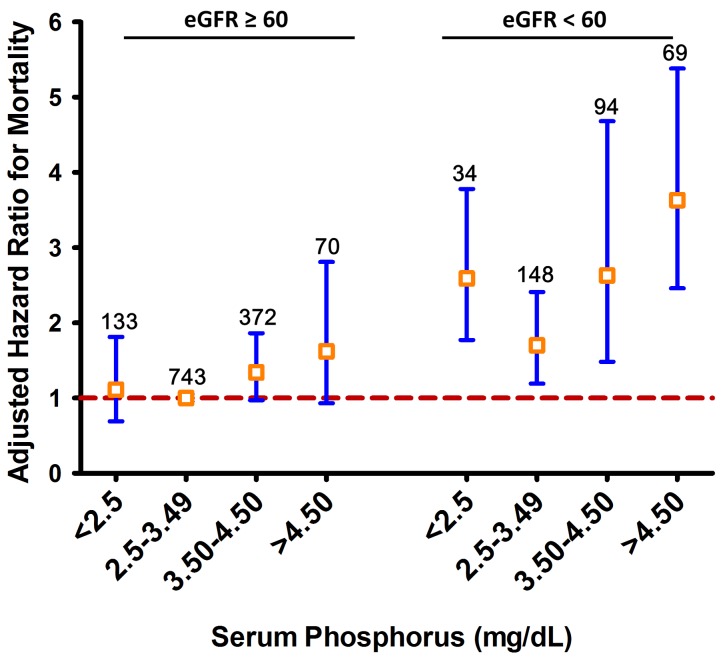
Combined effect of serum phosphorus and CKD. The figure shows adjusted hazard ratios and 95% confidence intervals for mortality according to serum phosphorus categories and CKD. The reference group includes patients with serum phosphorus of 2.50 to 3.50 mg/dL and without CKD. The number of patients in each group is shown above each bar.

We also studied whether the relationship between serum phosphorus and mortality varied according to renal function. The study population was divided into 8 groups based on serum phosphorus categories (<2.50–3.49, 3.50–4.5, >4.5) and presence or absence of CKD. Cox regression analysis based on these 8 groups demonstrated that the increased risk for mortality in patients with elevated serum phosphorus level occurred mainly in patients with CKD (Figure 4). Within the group of patients with CKD (n = 345), the hazard ratio for mortality in patients with serum phosphorus >4.5 mg/dL compared to patients with serum phosphorus 2.50–3.50 was 2.34 (95% CI 1.55–3.54; *P*<0.0001), whereas in patients without CKD (n = 1318), the HR for mortality associated with the presence of serum phosphorus >4.5 mg/dL was lower (HR 1.53, 95% CI 0.87–2.69; *P* = 0.14). Likelihood ratio tests demonstrated a significant interaction between serum phosphorus level and CKD with respect to mortality in an unadjusted model containing only the main effects of serum phosphorus and CKD (*P = *0.022) but not in the adjusted model (*P = *0.36).

### Change in Serum Phosphorus during Hospital Course

During hospital stay, serum phosphorus level increased from 3.4±0.9 mg/dL (measured at the morning of the first hospital day) to peak level of 3.9±1.1 mg/dL (*P*<0.0001). After adjustment for other factors independently associated with mortality including baseline phosphorus levels, an increase in serum phosphorus levels was associated with increased risk of mortality (adjusted hazard ratio 1.14 per 1-SD increase [95% CI 1.08 to 1.21, *P*<0.0001).

## Discussion

The present study demonstrates a graded, independent association between serum phosphorus level and adverse clinical outcome in patients after AMI. The association was robust for the endpoints of incident all-cause mortality and heart failure. A higher risk for mortality was present even for serum phosphorus levels within the normal range. However, the association between serum phosphorus levels and mortality was more prominent in the presence of CKD.

In univariable analyses, the associations of phosphorus levels with the clinical outcome were J-shaped, with higher risks observed also in patients with hypophophatemia. These finding are consistent with the known effects of low phosphorus levels on the cardiovascular system, such as decreased contractility and arrhythmias [20], [21]. However, after adjustment for the relevant comorbid conditions, lower levels of serum phosphorus were no longer associated with increased mortality risk.

Blood level of phosphorus are affected by intake, output, and intracellular shift, with multiple regulatory factors at the level of intestinal absorption and excretion, flux into and out of bone, glomerular filtration, and tubular reabsorption [22], [23]. In the present study, the relationship between serum phosphorus and renal function was biphasic, with no association between phosphorus and renal function for eGFR above 60 ml/min/1.73 m**^2^** and a linear increase in serum phosphorus for eGFR levels below 60 ml/min/1.73 m**^2^**. However, elevated phosphorus levels were also present in patients with preserved renal function. These findings are consistent with previous studies demonstrating that only a small portion (∼12%) of the variation in serum phosphorus concentrations could be explained by kidney function, cardiovascular risk factors, diet, and demographics [24]. Thus, factors determining serum phosphorus concentration are largely unknown, and the observed association of serum phosphorus concentrations with all-cause mortality and heart failure are unlikely to reflect differences in dietary intake or traditional cardiovascular risk factors [24].

Vascular calcification (especially when levels of calcium-phosphorus product are high) has been implicated as a potential mechanism for the association between elevated phosphorus levels and adverse cardiovascular outcomes [25], [26]. Vascular calcification is not merely a function of an elevated calcium-phosphorus product, but a complex process regulated by several mediators, including fibroblast growth factor 23 (FGF-23), and the crystallization inhibitors uncarboxylated matrix Gla protein (ucMGP), inorganic pyrophosphate, and fetuin-A [27]–[32]. The hallmark of vascular calcification is calcium phosphate deposition, which can occur in the vasculature, myocardium, and cardiac valves. High phosphorus concentrations promote non-atherosclerotic arterial calcification by stimulating vascular smooth muscle cells (VSMCs) to transform from a contractile phenotype into an osteochondrogenic phenotype that promote mineralization of surrounding arterial tissue [25], [33]. These phosphate-induced phenotypic changes in VSMCs appear to be dependent on the activity of sodium-dependent phosphate cotransporter PiT-1 [25], [34], [35]. Vascular calcification occur independently of atheroma and are an important cause of vascular stiffness leading to increased pulse wave velocity, increased cardiac work and left ventricular hypertrophy [25], [36]–[39].

Cardiovascular mortality in CKD patients with hyperphosphatemia has been proposed to occur predominantly through arrhythmic mechanisms on a background of a susceptible myocardial substrate due to hypertrophy/fibrosis resulting from arterial stiffening [8], [36]. In the Framingham heart study, higher serum phosphorus was associated with greater left ventricular mass and larger left ventricular internal dimensions in individuals without prior myocardial infarction, and this association was independent of hypertension [7]. Furthermore, in chronic hemodialysis patients, sudden death accounted for the greatest proportion of all deaths associated with hyperphosphatemia [2]. This may explain the strong association of serum phosphorus with all-cause mortality and heart failure in the present study of patients after AMI, who are at high risk for both heart failure and arrhythmic events.

By contrast, it is unclear whether hyperphosphatemia predisposes to atherosclerotic plaque rupture [9], [36]. Thus, it is difficult to extrapolate the paradigm of hyperphosphatemic arterial medial calcification and arterial stiffening to the occurrence of acute myocardial infarctions. This may explain the weaker association of phosphorus levels with recurrent infarction and stroke in the present study. However, the association may also be confounded by coronary and cerebral events that lack a pathogenic link with vascular calcification such as periprocedural infarctions, stent thrombosis, and cerebral embolism due to atrial fibrillation and left ventricular thrombus.

Of note, similar heterogeneity with regard to the relationship between phosphorus and different cardiovascular outcomes has been reported in other studies. In 15,732 participants of the Atherosclerosis Risk in Communities Study with mean follow-up of 12.6 years, higher serum phosphorus was associated with increased risk for mortality but not of coronary disease [5]. An analysis of 7,259 postmenopausal women reported no associations between serum phosphorus and incident cardiovascular events during 4 years of follow-up [6]. In a recent nested case-control study of men without CKD from the Health Professionals Follow-up Study, phosphorus levels were not associated with the development of incident CAD during 10 years of follow-up [40] Collectively, these results suggest that the association between serum phosphorus level and different clinical endpoints may be dependent on the baseline clinical characteristics and risks of the study population.

Importantly, in some studies, the definition of incident CVD included congestive heart failure together with myocardial infarction, angina pectoris and cerebrovascular events [3], [41]. Analyzed this way, our data also shows a significant association between serum phosphorus levels and CVD events. However, stratification according to specific endpoints provided insight into the apparent discordance with previous investigations.

To date, few studies have examined associations between serum phosphorus levels and cardiovascular disease in populations with established coronary disease. One group, however, reported a positive trend between higher serum phosphorus levels and an increased risk of mortality and CVD events during a 5-year follow-up period in patients with a previous myocardial infarction [10].

Formal interaction testing concerning the influence of CKD on the serum phosphorus association with survival was not statistically significant in multivariable models. Notwithstanding, an effect modification between serum phosphorus and eGFR is biologically plausible, and may be explained by the fact that several inhibitors of the calcification process are dysregulated in patients with CKD [42], [43]. For example, the plasma concentrations of the inactive uncarboxylated form of matrix Gla protein are markedly increased in CKD stage 4 [44]. In CKD, circulating fetuin- A and pyrophosphate are reduced compared to healthy subjects [45], [46]. Thus, individuals with moderate or severe renal disease have an impaired ability to excrete phosphorus, and may be more susceptible to transient fluctuations in serum calcium and to post-prandial peaks in serum phosphorus [47].

### Study Limitations

Our study has important limitations that must be acknowledged. Parathyroid hormone, vitamin D and FGF-23, also associated with adverse outcomes [27] were not measured. The cross-sectional design does not allow causal inference. Although we adjusted for multiple potential risk factors, another limitation of this observational study is that we cannot rule out the possibility of residual confounding. Thus, there remains the possibility that unmeasured confounders might explain the observed associations of phosphorus with mortality and heart failure.

### Conclusions

We found a graded, independent association between serum phosphorus levels and all-cause mortality and heart failure in patients after AMI. The risk for mortality appears to increase with serum phosphorus levels within the normal range. Further studies are needed to determine the contributing mechanisms and clinical implications of the association between serum phosphorus and clinical outcome in the post-AMI setting.
